# Effects of Stressors of COVID-19 on Chinese College Students' Problematic Social Media Use: A Mediated Moderation Model

**DOI:** 10.3389/fpsyt.2022.917465

**Published:** 2022-06-30

**Authors:** Jun Zhao, Baojuan Ye, Li Yu, Fei Xia

**Affiliations:** ^1^Center of Mental Health Education and Research, School of Psychology, Jiangxi Normal University, Nanchang, China; ^2^Mental Health Education and Counseling Center, Nanchang Hangkong University, Nanchang, China; ^3^School of Education, Nanchang Institute of Science and Technology, Nanchang, China

**Keywords:** stressors of COVID-19, Chinese college students, problematic social media use, fear of missing out, regulatory emotional self-efficacy

## Abstract

**Purpose:**

Isolation policies are long-term and strictly enforced in China during the COVID-19 outbreak. Social media might be widely used for communication, work, understanding the development of the epidemic, etc. However, these behaviors might lead to problematic social media use. The present study investigated the effect of stressors of COVID-19 on problematic social media use, as well as the internal mechanisms involved.

**Methods:**

One thousand three hundred seventy-three Chinese college students (*M*_age_ = 19.53, *SD*_age_ = 1.09) were recruited randomly from four grades who completed Coronavirus Stress Scale, Fear of Missing Out Scale, Problematic Mobile Social Media Usage Assessment Questionnaire, and Regulatory Emotional Self-Efficacy Scale.

**Results:**

Stressors of COVID-19 were positively related to problematic social media use. The link between stressors of COVID-19 and problematic social media use was mediated by fear of missing out. Additionally, the association between fear of missing out and problematic social media use, as well as the association between stressors of COVID-19 and problematic social media use were moderated by regulatory emotional self-efficacy.

**Conclusion:**

The current findings reveal the mechanism that may be used to reduce the likelihood of problematic social media use in the context of the COVID-19 outbreak. To prevent and intervene in problematic social media use during the COVID-19 pandemic, this study stressed the importance of decreasing the fear of missing out and enhancing regulatory emotional self-efficacy.

## Highlights

- Stressors of COVID-19 were positively related to smartphone problematic social media use.
- The effect of stressors of COVID-19 on smartphone problematic social media use was mediated by fear of missing out.
- The effect of fear of missing out on smartphone problematic social media use was moderated by regulation emotional self-efficacy. The effect of stressors of COVID-19 and smartphone problematic social media use was moderated by regulation emotional self-efficacy.

## Introduction

COVID-19 has caused significant negative consequences for the global economy, culture, life and public health (1, 2). Due to the COVID-19 pandemic, authorities implemented rigorous measures such as isolation to diminish infection rates (3, 4). Isolation policies that are long-term and strictly enforced may lead to significant changes in how adolescents engage socially (5, 6). Due to social distancing, smartphone social media use has increased for gathering epidemic information, studying, working, alleviating boredom, and social networking online (7, 8). Social media is an essential tool in daily life during the COVID-19 epidemic (9, 10).

However, excessive social media use can be harmful. There is a sense of withdrawal when the user temporarily leaves the online world (11, 12). Then, improper social media usage may increase the likelihood of addiction during the epidemic (13, 14). Since the classification of problematic Internet use is conceptually unclear, there is a lack of consensus among academics on the definition of problematic social media use (15, 16). In comparison, addiction-like symptoms and everyday life disruptions have been attributed to the use of social media which is characterized by “addiction-like” behavior and exacerbates disputes with family and friends (17, 18). Most scholars recognize that problematic social media use refers to individuals spending a significant amount of time on social media, resulting in impairment of the individual's social, physical and mental health (11, 19, 20). Increasing numbers of studies from various countries describe its potential negative consequences that cannot be ignored. For instance, researchers revealed that problematic use of social media was positively correlated with depression and insomnia (21). Furthermore, Al-Menayes (22) found that social media addiction harmed academic performance. Moreover, the research revealed that Facebook addiction predicted suicide ideation and behavior (23). Kaye (24) found that physical health was linked to excessive social media usage. As a result, although prior researchers have discovered the detrimental consequences of problematic social media use, little research was undertaken among Chinese college students during the epidemic, which is one of the study's focal focuses.

### Stressors of COVID-19 and Problematic Social Media Use

Stress is caused by an inability to meet the multiple demands of one's environment, which may overwhelm an individual's capacity to adapt (25). Although keep social distancing played a very important role during the epidemic, it also negatively affected the individual's social activities. Due to the lack of effective medication for COVID-19, anxiety, and depression symptoms accompany a loss of control over an individual's daily activities (4, 26). Individuals suffered severe stress during COVID-19 (27, 28). Lazarus and Folkman distinguished two kinds of strategies in people's coping with stress: problem-focused coping and emotion-focused coping (29). One included an attempt to resolve the stress(itself)-causing the problem, while the other described people's attempts to manage the emotions that are prompted by stress. During the epidemic, people might obtain COVID-19 related information *via* social media with problem-focused coping strategies which could keep abreast of COVID-19 (8, 9). In addition, people might join online communities for social support to cope with their negative feelings caused by the pandemic (7, 30). Nevertheless, it's important to take into account the potentially harmful effects of problematic social media use through some online activities (17, 31). Previous researches showed the problematic use of SNS was favored by unmet need to belong and anxious attachment style (32, 33). Recent studies were conducted before the COVID-19 pandemic which had found daily stress had a significant effect on addictive social media (12, 34, 35). Therefore, we proposed that Chinese college students who experienced higher stressors of COVID-19 might be at higher risk of problematic social media use.

### The Mediation Effect of Fear of Missing Out

According to stress-coping strategies (29), college students are subjected to stressors of COVID-19, which may lead to an increase in problematic social media use. Simultaneously, other psychological variables may also have a significant influence on the relationship between stress and problematic social media use. Then, while examining the impacts of stressors of COVID-19, it is critical to examine the mediators that influence the emergence of problematic social media use. Fear of missing out is characterized as a constant fear that others may be enjoying gratifying experiences that one is not a part of, and the urge to keep up with what others are doing (36). The fear of missing out has grown ubiquitous in popular society (37, 38). Eighty-one percent of participants in recent research of 936 people from various socio-demographic backgrounds reported experiencing fear of missing out at least periodically (39). Previous studies have found that the association between negative factors and social media engagement was mediated by fear of missing out (36). Meanwhile, fear of missing out was an important risk factor for social media engagement (37, 40). Earlier researches addressed fear of missing out as a trait variable, but more academic work has examined the degree to which environmental signals, such as checking social media postings, might cause state- fear of missing out (41). When individuals utilize digital tools to keep track of tantalizing things on social media about desirable online activities, they may get state-fear of missing out (42, 43). In this study, the variable we investigate is the state- fear of missing out. In contrast to previous studies, our study focuses on determining how fear of missing out has evolved during the COVID-19 epidemic when individuals reduced their outside activities in favor of online activities. People keep social distance to prevent the spread of COVID-19, which is an effective measure. However, the unique features of COVID-19 make it an exceptional situation, with concomitant anxiety and depressive symptoms accompanied by a loss of social support (i.e., control over daily life and offline social interaction) (28, 44). With chronic psychological deficits, people are continually looking for updates through social media (9, 45). According to the social compensation theory (46), individuals seeking online support are motivated by a lack of offline social support. Previous researchers found that online social media could remind individuals that they might have missed otherwise (16, 43). During the epidemic, people mainly use smartphone social media to obtain information about the epidemic and conduct social activities to reduce negative emotions (27). With constant access to online social media, individuals increasingly expect and await feedback. Then, individuals are afraid of missing out on this instant information related to COVID-19 from authority and feedback from their friends (16, 42), which may lead to a higher level of fear of missing out during COVID-19. Fear of missing out includes unmet social needs and has symptoms of depression and anxiety (41, 42, 45). In conformity with the compensatory Internet use theory (47), when individuals experience negative events or emotions, they turn to the Internet to relieve these confusions, leading to Internet overuse. During the pandemic of COVID-19, pandemic-related social distancing and limiting public gatherings led to fewer socialization options. When people were under stressors, they often unconsciously unlocked their phones and used online social media (40, 48), which might increase problematic social media use (18, 35). As a result, stressors of COVID-19 are associated with an increased risk of problematic social media use in those who use social media to alleviate their fear of missing out on meeting their social support requirements. We hypothesized in this research that the association between stressors of COVID-19 and Chinese college students' problematic social media use was mediated by fear of missing out.

### The Moderating Role of Regulatory Emotional Self-Efficacy

A stressor will not have a detrimental effect on those with adequate coping resources, according to stress-coping strategies (16, 29, 49). To put it another way, a person's behavior is not just determined by the amount of stress they experience but also by how effectively they are able to manage that stress (50). Researchers found that the ability of stress-coping moderated the relationship between stressful life events and a wide range of developmental outcomes, including substance use, externalizing problems, and internalizing problems (51–53). During the epidemic, not all individuals who experience stressors of COVID-19 increase their fear of missing out and suffer from problematic social media use. The heterogeneity of outcomes may reflect individual characteristics (54) that moderate (i.e., protect) the effect of stressors of COVID-19 on problematic social media use. One such protective factor may be regulatory emotional self-efficacy.

Regulatory emotional self-efficacy refers to a person's perceived degree of confidence in their capacity to manage emotions (55, 56). In accord with the self-efficacy theory (57), individuals only have the motivation to carry out the activity if they believe that the prospective result can be achieved. Previous studies have confirmed that regulatory emotional self-efficacy played a vital role in individuals' actual engagement in adaptive behavior (58, 59). Several previous findings have demonstrated that regulatory emotional self-efficacy was negatively related to addictive behavior, which plays a protective role (56–61).

The association between environmental risk factors and problem behaviors would be reduced by individual attributes such as regulatory emotional self-efficacy, in line with the “risk buffering hypothesis” (62–64). The hypothesis proposed that protective factors may mitigate the negative consequences of risk factors. Regulatory emotional self-efficacy may serve as a buffer in this study. As individuals feel more stress, psychological needs in their lives are not met, but individuals with higher levels of emotion management abilities leave individuals with lower needs for desired compensation which lead to less problematic social media use behaviors (65). Furthermore, as the fear of missing out rises, individuals in their lives are at a lower risk of tending to use mobile social media due to higher emotional management abilities (66). Then, potentially harmful results (problematic social media use) might be reduced by the combination of protective (regulatory emotional self-efficacy) and risk (stressors of COVID-19, fear of missing out) variables that interact. The “risk buffering hypothesis” is confirmed by empirical researches. For instance, Guo et al. (67) found that regulatory emotional self-efficacy played a moderated role in the association between parental psychological control and adolescent non-suicidal self-injury. Besides, the previous research also revealed that the link between emotional reactivity and suicide ideation was buffered by regulatory emotional self-efficacy (59). Meanwhile, Pan et al. (68) found that individuals with high levels of regulatory emotional self-efficacy have a lower risk of Internet addiction. Taken together, we predicted that regulatory emotional self-efficacy moderated the effect of stressors of COVID-19 on problematic social media use, as well as the effect of fear of missing out on problematic social media use.

### The Present Study

**Figure 1:**
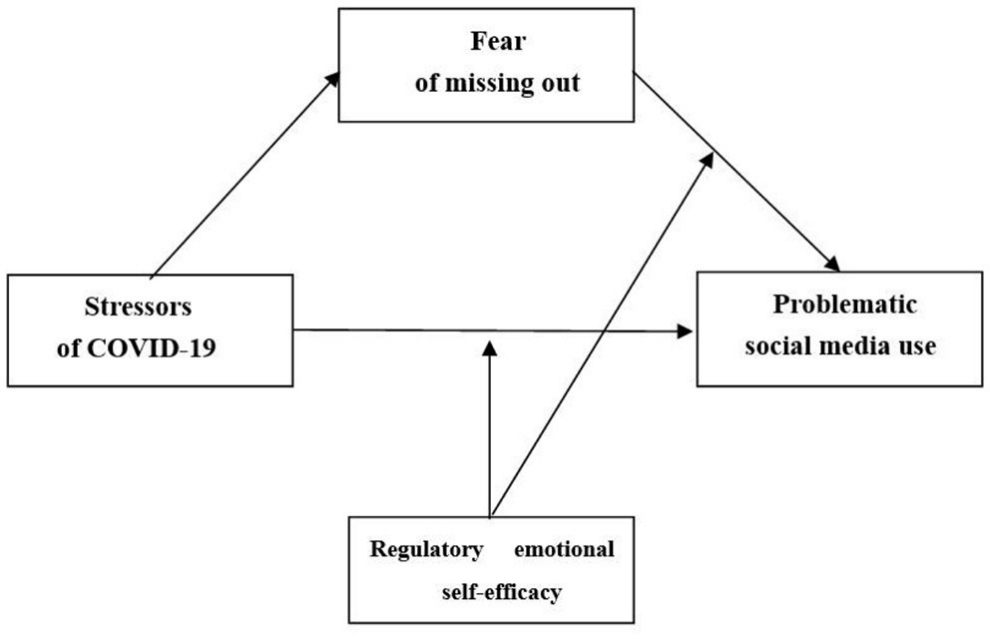
Conceptual model.

To summarize, we developed a moderated mediation model to address three questions: (a) whether stressors of COVID-19 would be linked with problematic social media use, (b)whether the effect of stressors of COVID-19 on problematic social media use would be mediated by fear of missing out, (c)whether regulatory emotional self-efficacy would moderate the effect of fear of missing out on problematic social media use as well as the effect of stressors of COVID-19 on problematic social media use. The moderated mediation model was outlined in Figure 1. We put forward three hypotheses:

**Hypothesis 1:** Stressors of COVID-19 are positively correlated with problematic social media use.

**Hypothesis 2:** The effect of stressors of COVID-19 on problematic social media use would be mediated by fear of missing out.

**Hypothesis 3:** The effect of fear of missing out on problematic social media use would be buffered by regulatory emotional self-efficacy. The effect of stressors of COVID-19 on problematic social media use would be buffered by regulatory emotional self-efficacy.

## Method

### Participants

One thousand four hundred two Chinese college students were recruited in China. The criteria for unqualified participants were <100 s to complete questionnaires with a total of 47 questions and regularity of answers, such as the same score in each item or a regular pattern of scores (1, 2, 3, 4, 5, 1, 2, 3, 4, 5, 1, 2, 3, 4, 5, etc.). After excluding unqualified samples (e.g., completed questionnaire <100 s and answered regularly), we finally collected 1,373 (*M*_age_ = 19.53, *SD*_age_ = 1.09) valid questionnaires with an effective response rate of 97.93% from 1,402 primary questionnaires. The mean age ranges from 18 to 23 years. 56.67% of participants were females. Regarding their grades, 26.91% were freshmen, 21.15% were sophomores, 29.31% were junior students, and 22.63% were senior students.

### Instruments

#### The Coronavirus Stress Scale

We used the 5-item Coronavirus Stress Scale (69) to measure individuals' perceived COVID-19 related stress. All items (e.g., How often have you felt that you were unable to control the important things in your life due to the COVID-19 pandemic?) were rated on a five-point scale (from 1 = never to 5 = very often). Applied to Chinese samples, the cultural adaption and reliability of the revised scale are well (70, 71), and the validity of the revised scale is well (71–73). The goodness of fit [CFI = 0.91, TLI = 0.91, RMSEA = 0.08, 90% *CI* = (0.07, 0.09)] of confirmatory factor analysis of the Coronavirus Stress Scale in this study indicated that fit indicators in accordance with the cutoffs recommended in the literature actually showed a satisfactory adequacy, indicating that the scale had good construct validity. In this study, Cronbach's α for the scale was 0.90.

#### Fear of Missing Out Scale

We used the 10-item Fear of Missing Out Scale (36) to assess the missing out on social events and spending time with friends. Each item (e.g., I fear that my friends have more rewarding experiences than me.) was rated on a five-point scale (from 1 = Not at all true of me” to “5 = Extremely true of me). Applied to Chinese samples, the cultural adaption and reliability of the revised scale are well (40, 74, 75), and the validity of the revised scale is well (74, 76). The goodness of fit [CFI = 0.92, TLI = 0.91, RMSEA = 0.08, 90% *CI* = (0.07, 0.09)] of confirmatory factor analysis of the Fear of Missing Out Scale in this study indicated that that fit indicators in accordance with the cutoffs recommended in the literature actually showed a satisfactory adequacy, indicating that the scale had good construct validity. In this study, Cronbach's α for the scale was 0.89.

#### Problematic Mobile Social Media Usage Assessment Questionnaire

We used the 20-item Problematic Mobile Social Media Usage Assessment Questionnaire (77) to evaluate problematic smartphone social media usage. All the items (e.g., If you are delayed in doing business due to using social networks, you often regret that you have lost your time by playing on your mobile phone.) were rated on a five-point scale (1 = never to 5 = very often). Applied to Chinese samples, the cultural adaption and reliability of the revised scale are well (20, 78, 79), and the validity of the revised scale is well (20, 80). The goodness of fit [CFI = 0.93, TLI = 0.92, RMSEA = 0.07, 90% *CI* = (0.06, 0.08)] of confirmatory factor analysis of the Problematic Mobile Social Media Usage Assessment Questionnaire in this study indicated that fit indicators in accordance with the cutoffs recommended in the literature actually showed a satisfactory adequacy, indicating that the scale had good construct validity. In our study, Cronbach's α for the scale was 0.89.

#### Regulatory Emotional Self-Efficacy Scale

We used the 12-item Regulatory Emotional Self-Efficacy Scale (55) to measure the individual's confidence in the ability to regulate their own emotions. Responses to each item (e.g., Keep from getting discouraged in the face of difficulties?) were rated on a five-point scale (1 = not well at all to 5 = very well). Applied to Chinese samples, the cultural adaption and reliability of the revised scale are well (59, 61, 67), and the validity of the revised scale is well (81, 82). The goodness of fit [CFI = 0.92, TLI = 0.91, RMSEA = 0.07, 90% *CI* = (0.06, 0.08)] of confirmatory factor analysis of the Regulatory Emotional Self-Efficacy Scale in this study indicated that fit indicators in accordance with the cutoffs recommended in the literature actually showed a satisfactory adequacy, indicating that the scale had good construct validity. In our study, Cronbach's α for the scale was 0.92.

### Procedure

The study was approved by the ethical committee of the first author's University. Before data collection, participants' consent was acquired. Questionnaires were delivered online Internet to comply with the epidemic prevention policy. All replies were anonymous, and all questionnaires had detailed instructions. Participants were not compensated in any way for their involvement in the research.

### Analytic Approach

The data obtained from the normality tests revealed that none of the research variables deviated significantly from normalcy (83) (i.e., Skewness < |3.0| and Kurtosis < |10.0|). This study used the methods for estimating multivariate normality which was explored in terms of calculating Mahalanobis distances and plotting them on a scattergram against derived chi-square values using Fortran and SPSS programs developed by Thompson (84). If the variables form a multivariate normal distribution, the points will form a straight line (85). The result indicated that that multivariate normality can be assumed. Descriptive statistics were the first to be computed. The PROCESS Models 4 and 15 macros for SPSS were used to analyze the mediation and moderated mediation models using 5,000 random sample bootstrapping confidence intervals (CIs). A thorough standardization procedure was followed before the data analysis.

## Results

### Preliminary Analyses

The descriptive statistics and bivariate correlation of the relevant variables are shown in Table 1. Stressors of COVID-19 were positively associated with fear of missing out and positively associated with problematic social media use. Fear of missing out was positively associated with problematic social media use. Regulatory emotional self-efficacy was negatively associated with problematic social media use. As expected, the findings were in line with Hypothesis 1.

**Table 1:** Descriptive statistics and correlation matrix for the main variables.

	***M***	***SD***	**1**	**2**	**3**	**4**	**5**
1. Age	19.53	1.09	1				
2. SOC	2.49	0.87	0.16	1			
3. FOMO	2.41	0.88	−0.04	0.53**			
4. PSMU	2.39	0.87	0.02	0.57**	0.75**		
5. RESE	3.31	0.69	−0.03	−0.06	0.25	−0.08**	1

*N = 1,373,*

** *p < 0.01; SOC, Stressors of COVID-19; FOMO, Fear of missing out; PSMU, Problematic social media use; RESE, regulatory emotional self-efficacy*.

### Testing the Mediating Role of Fearing of Missing Out

In the hypothesis, we anticipated that the association between stressors of COVID-19 and problematic social media use was mediated by fear of missing out. Model 4 of Hayes' SPSS macro PROCESS was used to test this hypothesis (86). Table 2 shows the results of the regression analysis conducted to test mediation. Specifically, stressors of COVID-19 were shown to be positively correlated with fear of missing out, *β* = 0.52, *p* < 0.001, 95% *CI* [0.47, 0.57] and problematic social media use, *β* = 0.56, *p* < 0.001, 95% *CI* [0.53, 0.61]. Stressors of COVID-19 had a positive residual direct effect on problematic social media use. The results demonstrated that the association between stressors of COVID-19 and problematic social media use was mediated by fear of missing out, indirect effect = 0.32, *SE* = 0.02, 95% *CI* = [0.28, 0.37]. The mediated impact accounted for 57.13% of the overall effect of stressors of COVID-19 on problematic social media use. Our hypothesis 2 was confirmed by the results.

**Table 2:** Linear regression models.

**Predictors**	**Dependent variable (FOMO)**		**Dependent variable (PSMU)**		**Dependent variable (PSMU)**		**Dependent variable (PSMU)**	
	**β**	***t***	**β**	***t***	**β**	***t***	**β**	***t***
Age	−0.11	−4.74	0.01	0.41	0.04	2.30	0.03	2.23*
SOC	0.52	23.02***	0.56	25.77***	0.24	12.11***	0.22	10.63***
FOMO					0.62	31.37***	0.66	30.38***
RESE							−0.05	−2.53*
SOC × RESE							0.04	2.55*
FOMO × RESE							−0.05	−3.47***
*R^2^*	0.29		0.33		0.61		0.62	
*F(df)*	186.97***		227.33***		538.88***		314.97***	
	(3, 1,369)		(3, 1,369)		(4, 1,368)		(7, 1,365)	

*N = 1,373;*

* *p < 0.05,*

*** *p < 0.001; SOC, Stressors of COVID-19; FOMO, Fear of missing out; PSMU, Problematic social media use; RESE, regulatory emotional self-efficacy*.

### Testing for Moderated Mediation

Model 15 in SPSS macro PROCESS was used to examine if regulatory emotional self-efficacy might moderate the direct link between stressors of COVID-19 and problematic social media use, and the mediation effect of fear of missing out, as expected in this study (in particular, the association between problematic social media use and the fear of missing out). Table 2 shows the findings.

The analysis revealed that stressors of COVID-19 were linked to problematic social media use [*β* = 0.22, *p* < 0.001, 95% *CI* (0.17, 0.26)], whereas the fear of missing out was linked to the problematic social media use [*β* = 0.66, *p* < 0.001, 95% *CI* (0.63, 0.71)]. Aside from that, the interaction of stressors of COVID-19 and regulatory emotional self-efficacy [*β*= 0.04, *p* < 0.05, 95% *CI* [0.01, 0.07]) for problematic social media use was found to be significant, as was the interaction of fear of missing out and regulatory emotional self-efficacy (*β* = −0.05, *p* < 0.001, 95% *CI* (−0.08, −0.02)] for problematic social media use. Regulatory emotional self-efficacy was shown to reduce the links between stresses of COVID-19 and fear of missing out on problematic social media use (i.e., the relationship with stressors of COVID-19 and problematic social media use, as well as the relationship between fear of missing out and problematic social media use, might be significantly moderated by regulatory emotional self-efficacy). Consequently, the postulated moderated mediating model was shown to be well.

**Figure 2:**
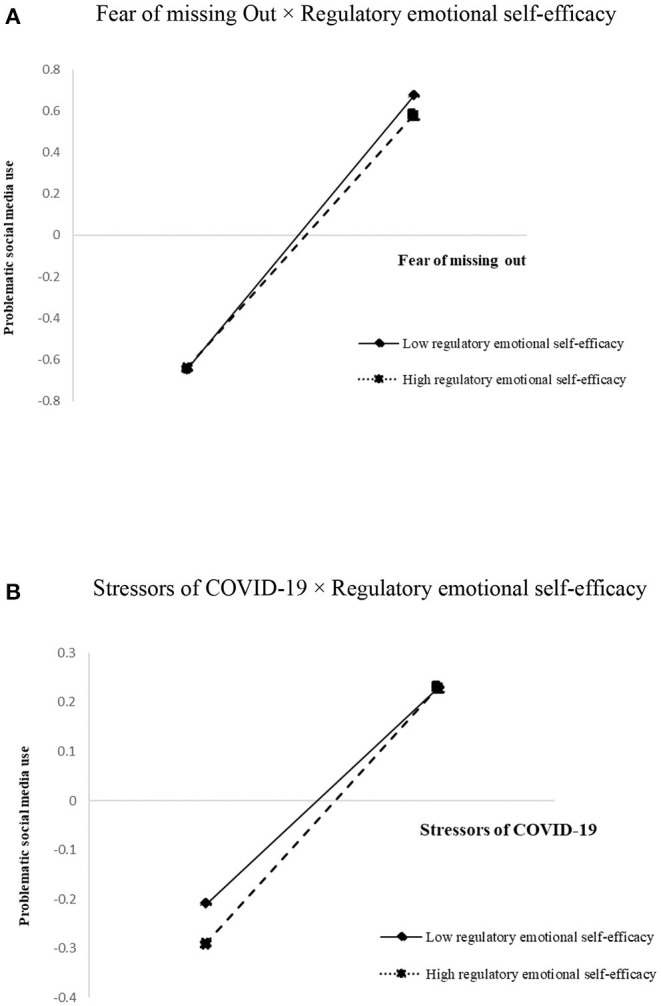
The plot of the relationship between fear of missing out and problematic social media use at two levels of regulatory emotional self-efficacy **(A)**. The plot of the relationship between stressors of COVID-19 and problematic social media use at two levels of regulatory emotional self-efficacy **(B)**. **(A)** Fear of missing Out × Regulatory emotional self-efficacy. **(B)** Stressors of COVID-19 × Regulatory emotional self-efficacy.

Figure 2 depicts a graphic representation of the interaction effect. In college students with low regulatory emotional self-efficacy, fear of missing out was shown to be a strong effect on problematic social media use, as demonstrated by simple slope tests, *b*_*simple*_ = 0.71, *t* = 23.34, *p* < 0.001. Fear of missing out, on the other hand, was shown to be a significant effect on problematic social media use among college students who had high regulatory emotional self-efficacy, but the relationship was considerably weaker, *b*_*simple*_ = 0.61, *t* = 27.56, *p* < 0.001, showing a buffering impact of regulatory emotional self-efficacy (Figure 2A). Finally, in Figure 2B, the visual representation of the interaction effect is shown. The results of simple slope tests revealed that stressors of COVID-19 significantly affected problematic social media use in both college students with high and low levels of regulatory emotional self-efficacy; however, for college students with high levels of regulatory emotional self-efficacy, the effect of stressors of COVID-19 on problematic social media use was stronger (*b*_*simple*_ = 0.26, *t* = 11.76, *p* < 0.001) than college students with low levels of regulatory emotional self-efficacy (*b*_*simple*_ = 0.18, *t* = 6.01, *p* < 0.001), demonstrating that regulatory emotional self-efficacy acted as a buffer in the opposite direction.

Further comparing the indirect effects, the difference between a high degree of regulatory emotional self-efficacy and a low degree of regulatory emotional self-efficacy also reached a significant level [Index of moderated mediation, Index = – 0.028, 95% *CI* = (−0.06, −0.02)], indicating the degree of regulatory emotional self-efficacy weakens the indirect effects of fear of missing out between stressors of COVID-19 and problematic social media use. The indirect effect of stressors of COVID-19 on problematic social media use *via* fear of missing out was further moderated by regulatory emotional self-efficacy, according to the bias-corrected percentile bootstrap analysis. The indirect impact of stressors of COVID-19 on problematic social media usage through fear of missing out was particularly significant for college students with low levels of regulatory emotional self-efficacy, *β* = 0.37, *SE* = 0.02, 95% *CI* = [0.33, 0.43]. The indirect effect was likewise significant for college students who had strong refusal self-efficacy, but was lower for those who had weak refusal self-efficacy, β = 0.32, *SE* = 0.02, 95%*CI* = [0.27, 0.37]. As a result of the findings, hypothesis 3 was supported by all two moderating routes shown in Figure 1.

## Discussion

In this study, we explored the effects of stressors of COVID-19 on Chinese college students' problematic social media use. The results suggested that stressors of COVID-19 were positively related to problematic social media use. Furthermore, the effect of stressors of COVID-19 on problematic social media use was mediated by fear of missing out. The effect of fear of missing out on problematic social media use was moderated by regulatory emotional self-efficacy, as well as the effect of stressors of COVID-19 on problematic social media use. Then, we had a clear grasp of how and when stressors related to COVID-19 were associated with problematic social media use.

The findings revealed that stressors of COVID-19 were closely correlated with problematic social media use. It meant that college students who experienced a higher level of stressors of COVID-19 were more likely to engage in problematic social media behavior. The result was consistent with the general strain theory (87), which proposes that different types of stress cause individuals' negative experiences that ultimately lead to problematic behaviors. During the epidemic, effects of the absence of specific drugs for COVID-19 and the reduction in social interaction due to keeping a social distance (4) predispose individuals to develop negative emotions (26). As epidemic prevention measures were strictly enforced, access to social interactions decreased and mobile phone use increased (64), with the consequently increased availability of mobile social media use (16, 44). Through the use of social media, individuals were able to obtain positive social feedback and momentarily forget about negative emotions (21, 88). Therefore, such a process increased the risk of problematic social media use.

### The Mediation Role of Fear of Missing Out

Our study showed that fear of missing out mediated the association between stressors of COVID-19 and problematic social media use. Then, in light of stressors of COVID-19, fear of missing out might be one of the explaining mechanisms for why some individuals are more prone to raise problematic social media use.

In the mediation process of the relationship between stressors of COVID-19 and the fear of missing out, stressors of COVID-19 have raised the fear of missing out among college students, which is consistent with social compensation theory (46). Individuals with high levels of stressors of COVID-19 are more likely to use social media to access positive psychological experiences that are not available in the context of isolation (16, 89). Individuals pay more attention to the information in the network with higher stressors of COVID-19 (14, 34, 90), and are more worried about missing important information about themselves or others in social media (42). In the mediation process of the relationship between fear of missing out and problematic social media use, college students with higher fear of missing out are more likely to exhibit problematic social media use. This finding of the study conforms with the compensatory Internet use theory (47). A previous study found that individuals with a higher level of fear of missing out required the psychological need to stay informed and avoid missing out on the experiences, thoughts, and experiences of others (91). This psychological need is greatly satisfied through the use of smartphone social media (1), and this psychological comfort also creates a sense of physical excitement in the individual (92), which can easily lead to social media overuse (45, 93).

### The Moderation of Regulatory Emotional Self-Efficacy

The association between fear of missing out and problematic social media use was moderated by regulatory emotional self-efficacy as well as the association between stressors of COVID-19 and problematic social media use. There are two different types of protection: risk-buffering and reverse risk-buffering (94). Regulatory emotional self-efficacy functioned as a buffer to the effect of college students' fear of missing out on problematic social media use. Fear of missing out on problematic social media use is mitigated by regulatory emotional self-efficacy. During the pandemic, individuals with high regulatory emotional self-efficacy are less likely to resort to problematic social media use for psychological satisfaction. The reason is that they can successfully manage their emotions (66) even when they are experiencing high fear of missing out. Individuals with high regulatory emotional self-efficacy, even if they suffer anxiety, fear, and other emotions, are more confident to deal with these negative emotions (95, 96). Therefore, our study results suggest that examining the “risk buffering hypothesis” (62) is crucial to understanding how college students' problematic social media use is impacted by the stressors of COVID-19.

In contrast, the effect of stressors of COVID-19 on smartphone problematic social media use is stronger for college students with high regulatory emotional self-efficacy than for those with a low level of regulatory emotional self-efficacy. Accordingly, the benefits of regulatory emotional self-efficacy are negated in the presence of high stressors of COVID-19 based on our findings. Stressors of COVID-19 can induce negative emotions (1), and the anonymity, convenience, and escapist nature of smartphone social media can provide individuals with relief and escape from these negative emotions (13, 97), prompting individuals to use mobile social media to cope with stress. People who have high regulatory emotional self-efficacy are more confident in their abilities, so they may appear to overrate themselves and tend to fall into blind optimism, and overestimate their abilities (55, 98, 99) that is typical of overconfidence (100, 101). Overconfidence among college students may lead to cognitive biases such as “superiority,” the illusion of control, and over-optimism in smartphone use. Thus, if college students with high regulatory emotional self-efficacy who are under a high level of stressors of COVID-19 overestimate their sense of control when using their smartphones or underestimate the negative consequences of excessive smartphone social media use, regulatory emotional self-efficacy may aggravate their smartphone problematic social media use. The risk mitigation capacity of the protective factor may deteriorate when risk variables rise to an excessive degree. The result is in line with the protective-limiting hypothesis (杯水车薪) (102). The hypothesis proposed that in the presence of a high-risk factor, the preventive effects of the protective factor are diminished. Researchers have sufficiently supported the protective-limiting hypothesis in explaining the moderating effect (64, 103–105).

### Implication and Limitations

The results of this study have implications for both theory and practice. Theoretically, as a follow-up to prior research, this study emphasized the mediating function served by fear of missing out, as well as the moderating role played by regulatory emotional self-efficacy amid the epidemic, respectively. The effect of stressors of COVID-19 on college students' smartphone problematic social media use was little explored before the outbreak of COVID-19. Social compensation theory, compensatory Internet use theory, and self-efficacy theory are all supported by this study's findings, which give empirical proof to the prior research. Practically, in the context of the epidemic, our findings have crucial implications for preventing and intervening with smartphone problematic social media use among college students. College students are at an important period in their academic, cognitive and physical, and mental development. They are more emotionally sensitive and vulnerable to stressful life events (106). colleges and families should provide measures to increase the satisfaction of their needs. For example, the college could carry out various activities, cultivate good interests and hobbies, increases social channels, etc., so that college students could meet their own needs. College students could face up to and accept their emotions and reinterpret the significance of events in some epidemic situations. With the reduction of negative emotions caused by stressors of COVID-19, it is possible to reduce the risk of problematic social media use.

There are certain limits to the current research that should be mentioned. Firstly, a cross-sectional design was used, which makes it impossible to infer causality in this study. Future studies may use experimental and longitudinal methods to further clarify causality. Secondly, response bias may have influenced the findings in this investigation, as in any study that relied only on self-reported data for data collection. Future research may attempt to gather data from a broad number of informants to further explore the existing results. Thirdly, the sample is not very heterogenous regarding age. To draw even more generalizable conclusions, the findings need be reproduced with additional, more inclusive or even representative samples. Fourthly, we introduce the idea of online social support as a way to compensate social isolation when fear of missing out is presented. This would be a more conceptual robust option, and researchers may look into more protective factors such as emotional regulation self-efficacy, rather than only risk factors such as fear of missing out in the future study.

## Conclusion

In sum, fear of missing out is an important mediating component in the assessment of the effect mechanism of stressors of COVID-19 on problematic social media use, as shown by this study. It is recommended that future research take this variable into account more thoroughly. Moreover, regulation emotional self-efficacy is not always a defender of protective variables and a buffer against the risk factors. A lot of things need to be done for college students to get help which includes mental health courses, individual and group psychological counseling, as well as improving their emotion management skills so that college students couldn't use social media problematically.

## Data Availability Statement

The original contributions presented in the study are included in the article/supplementary files, further inquiries can be directed to the corresponding author.

## Author Contributions

JZ: writing—original draft. BY: supervision and project administration. LY and JZ: investigation and writing—review and editing. LY and BY: resources. FX: revision of the manuscript. All authors contributed to the article and approved the submitted version.

## Funding

This study was supported by the Jiangxi University Party Construction Research Project (20DJQN020), the National Natural Science Foundation of China (72164018), National Social Science Fund Project (BFA200065), Jiangxi Social Science Foundation Project (21JY13), Jiangxi' Key Research Base Project of Humanities and Social Sciences (JD20068), and Science and Technology Research Project of Jiangxi' Department of Education (GJJ200306).

## Conflict of Interest

The authors declare that the research was conducted in the absence of any commercial or financial relationships that could be construed as a potential conflict of interest.

## Publisher's Note

All claims expressed in this article are solely those of the authors and do not necessarily represent those of their affiliated organizations, or those of the publisher, the editors and the reviewers. Any product that may be evaluated in this article, or claim that may be made by its manufacturer, is not guaranteed or endorsed by the publisher.
